# Bevacizumab suppresses the growth of established non-small-cell lung cancer brain metastases in a hematogenous brain metastasis model

**DOI:** 10.1007/s10585-019-10008-z

**Published:** 2019-11-25

**Authors:** Chinami Masuda, Masamichi Sugimoto, Daiko Wakita, Makoto Monnai, Chisako Ishimaru, Ryo Nakamura, Mari Kinoshita, Keigo Yorozu, Mitsue Kurasawa, Osamu Kondoh, Kaname Yamamoto

**Affiliations:** 1 Product Research Department, Kamakura Research Laboratories, Chugai Pharmaceutical Co., Ltd., 200 Kajiwara, Kamakura, Kanagawa 247-8530 Japan; 2 Chugai Research Institute for Medical Science Co., Ltd., 200 Kajiwara, Kamakura, Kanagawa 247-8530 Japan

**Keywords:** Bevacizumab, Non-small-cell lung cancer, Internal carotid artery, Hematogenous brain metastasis model, Luciferase reporter

## Abstract

Brain metastases are common in patients with non-small-cell lung cancer (NSCLC). The efficacy of bevacizumab, an anti-vascular endothelial growth factor (VEGF) humanized antibody, has been demonstrated in patients with nonsquamous NSCLC. We established a transplantable NSCLC cell line (Nluc-H1915) that stably expresses NanoLuc® reporter and confirmed the correlation between total Nluc activity in tumor and tumor volume in vivo. SCID mice inoculated with these cells through the internal carotid artery formed reproducible brain metastases, in which human VEGF was detected. Next, after metastases were established in the model mice (15–17 days), they were intraperitoneally administered weekly doses of human immunoglobulin G (HuIgG) or bevacizumab. Nluc activity in the brain was significantly lower in bevacizumab-treated mice than in HuIgG-treated mice. Additionally, bevacizumab concentration in the brain was higher in mice with brain metastasis than in normal mice, and bevacizumab was primarily observed in brain metastasis lesions. The microvessel density in brain metastasis was lower in bevacizumab-treated mice than in HuIgG-treated mice. We believe bevacizumab’s anti-proliferative effect on brain metastasis is due to anti-angiogenic activity achieved by its penetration into brain metastases; this suggests that a bevacizumab-containing regimen may be a promising treatment option for patients with NSCLC brain metastasis.

## Introduction

Patients with advanced non-small-cell lung cancer (NSCLC) commonly develop brain metastases; this occurs in 30–64% of the patients [1] and results in very poor prognosis. Whereas the survival of individuals with general metastatic NSCLC is reported to be approximately 12 months, the median survival for patients with brain metastases receiving palliative radiotherapy ranges from 2.4 to 4.8 months [2].

For patients with NSCLC with brain metastases that are symptomatic, the basic treatment approaches are radiation therapy and surgical resection, under the condition that all extracranial lesions are controlled [1]. On the other hand, when brain metastases in these patients are asymptomatic, systemic chemotherapies (platinum-based chemotherapy and/or molecular targeted drugs) are considered as treatment options, since they are expected to achieve a similar response intracranially as that seen extracranially [1]. However, the prognosis for survival of these patients continues to be poor, and further improvement is greatly needed.

Bevacizumab, a humanized anti-vascular endothelial growth factor (VEGF) monoclonal antibody (mAb), is approved, based on a high level of evidence, as first-line therapy for metastatic nonsquamous NSCLC in several countries around the world [3–7]. Clinical trials of bevacizumab in patients with recurrent glioblastoma multiforme (GBM) have reported a high response rate [8, 9]. Following this research on GBM, the studies BRAIN and EOLE, which investigated the use of bevacizumab in patients with nonsquamous NSCLC, demonstrated encouraging efficacy and an acceptable safety profile for chemotherapy that included bevacizumab as a first-line treatment for asymptomatic and untreated brain metastases [10, 11]. These results indicate that bevacizumab may be useful in patients with NSCLC with brain metastases. One of the specific obstacles to treating brain tumors may be the blood–brain-barrier (BBB), which is generally thought to limit the access of hydrophilic and/or large agents to the central nervous system, CNS [12, 13]. If so, the BBB blocks an antitumor drug from getting into the brain and targeting brain metastases [12–14]. Therefore, results of clinical studies need to be fully elucidated, specifically by showing that bevacizumab can penetrate the BBB, and by articulating a detailed mechanism for its antitumor effect on metastatic lesions.

Various models of brain metastasis have been reported, including the spontaneous model, the intracranial cellular engraftment model, and the hematogenous model, which respectively require intravenous, intracardial, or intracarotid injections of cancer cells [15]. Of these, the hematogenous model, which is improved by intracarotid inoculation, has been reported to produce brain metastases from various cancer cell lines efficiently and specifically [16, 17]. This model and others were used to investigate the role of VEGF signal in brain metastasis, and results suggest that bevacizumab and other VEGF-signaling blockade agents can prevent brain metastasis [16, 18]. However, it has not yet been confirmed whether bevacizumab has an antitumor effect on already existing brain metastases.

In the present study, we evaluated the antitumor efficacy of bevacizumab on established brain metastases in a xenograft mouse model of hematogenous brain metastases. Using the hematogenous model, which could approximate the status of the BBB seen in clinical settings, we investigated whether bevacizumab would be able to penetrate into metastatic lesions in the brain and exert an anti-angiogenic effect on the formation of tumor microvessels.

## Materials and methods

### Reagents

Bevacizumab was obtained from F. Hoffmann-La Roche Ltd. (Basel, Switzerland). Human immunoglobulin G (HuIgG) was purchased from MP Biomedicals (Santa Ana, CA, USA). Both bevacizumab and HuIgG were diluted with normal saline. Na-pyruvate and Geneticin™ Selective Antibiotic (G418 Sulfate) were purchased from Thermo Fisher Scientific, Inc. (Waltham, MA, USA). RPMI-1640 culture medium, d-glucose, and HEPES buffer were obtained from Sigma-Aldrich (St. Louis, MO, USA). Fetal bovine serum was obtained from Bovogen Biologicals Pty. Ltd. (Melbourne, Australia).

### Construction of pEBMulti-secNluc plasmid for reporter gene

The *Xba*I (blunt-ended)/*Kpn*I secreted NanoLuc® fragment [19] was removed from pNL2.3[secNluc/Hygro] (Promega Corporation, Madison, WI, USA) and ligated into the *Eco*RV/*Kpn*I sites of pEBMulti-Neo (Wako Pure Chemical Industries, Ltd., Osaka, Japan), resulting in the generation of the pEBMulti-secNluc plasmid.

### Cell lines and culture conditions

The human NSCLC cell line NCI-H1915, which was originally isolated from a brain metastasis [20], was obtained from American Type Culture Collection (Manassas, VA, USA) and maintained in RPMI-1640 medium supplemented with 10% fetal bovine serum, 4.11 g/l d-glucose, 10 mM HEPES buffer, and 1 mM Na-pyruvate at 37 °C in humidified air with 5% CO_2_. The Nluc-H1915 cell line, which stably expresses the luciferase reporter gene secNluc, was prepared by transfecting pEBMulti-secNluc plasmid into NCI-H1915 cells using FuGENE® HD Transfection Reagent (Promega Corporation), and then stable cells were selected and maintained in the culture medium containing 200 µg/ml of G418 Sulfate. The number of cells was measured using NucleoCounter® NC-200™ (ChemoMetec A/S, Allerod, Denmark).

### Laboratory animals

Seven-week-old male C.B-17/Icr-*scid*/*scid*Jcl mice and CB17/Icr-*Prkdc* < *scid* > CrlCrlj mice (SCID mice) were obtained from CLEA Japan, Inc. (Tokyo, Japan) and Charles River Laboratories Japan, Inc. (Kanagawa, Japan), respectively. All animal experiments were reviewed and approved by the Institutional Animal Care and Use Committee at Chugai Pharmaceutical Co., Ltd., and conformed to the Guide for the Care and Use of Laboratory Animals published by the Institute of Laboratory Animal Resources.

### Analysis of Nluc activity

Luminescence was measured using Nano-Glo® Luciferase Assay System (Promega Corporation), which provides a simple, single-addition reagent that generates a glow-type signal in the presence of NanoLuc® luciferase. The reagent was prepared by mixing Nano-Glo® Luciferase Assay Substrate with Nano-Glo® Luciferase Assay Buffer according to the manufacturer’s instructions. 2104 EnVision™ Multimode Plate reader (Perkin Elme, Inc., Waltham, MA, USA) was used to measure the luminescence.

### Evaluation of antitumor activity in a subcutaneous Nluc-H1915 model

SCID mice were subcutaneously inoculated with 5 × 10^6^ cells/mouse of Nluc-H1915 cells into the right flank and, when tumor volume (TV) reached 223–403 mm^3^, were randomized into control and test groups that received HuIgG and bevacizumab, respectively (day 1). TV was estimated from the equation TV = ab^2^/2, where a and b are tumor length and width, respectively. Bevacizumab or HuIgG was intraperitoneally administered once a week at a dose of 5 mg/kg (days 1, 8, and 15), based on the regimen in a previous report [21]. To evaluate the antitumor activity, TV and Nluc activity were measured on day 22. Nluc activity [relative light unit (RLU)/whole tumor] was measured in the supernatant of the tumor lysate, which was prepared by homogenizing the tumor specimen in a cell lysis buffer (Cell Signaling Technology, Inc., Danvers, MA, USA).

### Preparation of a brain metastasis model

To produce brain metastases, SCID mice were anesthetized by isoflurane and a microclamp was applied to the external carotid artery to prevent extracranial metastasis from forming. Then Nluc-H1915 cells (1 × 10^5^/50 µl RPMI-1640) were injected slowly into the internal carotid artery by surgical visualization, and finally the cut made in the skin was stitched up. Tumor inoculation was typically performed on 40 mice and approximately 10–30% of the mice died or were euthanized due to loss of locomotor activity before randomization. Approximately 10% of the mice didn’t develop brain metastasis based on an assessment of plasma Nluc activity and the rest of the mice were used for experiments.

After 15–17 days, the Nluc activity in plasma (RLU/5 µl) was measured and the plasma levels were used to randomize mice into control and test groups to obtain similar levels of average plasma Nluc activity in each group (day 1).　Then, 5 mg/kg of HuIgG and bevacizumab were administered intraperitoneally on days 1, 8, 15 into mice in the control and test groups, respectively. On day 21, the antitumor activity was evaluated by measuring the Nluc activity (RLU/whole brain) in the supernatant of the lysate of brain parenchyma homogenized with cell lysis buffer. The presence of brain metastases was examined under a microscope after hematoxylin and eosin (HE) staining or immunohistochemistry (IHC) with an anti-human EGFR antibody. The number of metastatic lesions having a maximum diameter of 300 µm or more was a minimum of 3 in our IHC analysis in control HuIgG-treated group.

### Protein extraction

Tumor tissues or brains were removed from mice on the specified day, frozen immediately in liquid nitrogen, and stored at − 80 °C until use. The tumor tissues or the brain parenchyma cells were homogenized in cell lysis buffer. The supernatant in each homogenate was collected after centrifugation and used for analyses.

### Measuring human VEGF and human IgG by enzyme-linked immunosorbent assay (ELISA)

After perfusion with saline, brains were removed and the concentrations of human VEGF and bevacizumab in the tissue samples of mice were determined according to the manufacturer’s instruction using a Human VEGF ELISA Kit (ab100663) and a HuIgG ELISA Kit (ab100547) (Abcam plc, Cambridge, UK), respectively. Benchmark Plus Microplate Reader (Bio-Rad Laboratories, Inc., Hercules, CA, USA) was used to detect the concentration of HuIgG in the tissue sample as an indication of the level of bevacizumab.

### Determining human EGFR and human IgG in tumor tissues by IHC

The mice were euthanized at the specified time points by exsanguination under anesthesia using isoflurane. Brains were removed without perfusion and sliced into six sections. Human NSCLC cells in the brain were stained by IHC with an anti-human EGFR antibody, EGFR (D38B1) XP® Rabbit mAb (Cell Signaling Technology, Inc.). Bevacizumab was stained by IHC using Polink-2 Plus HRP HuIgG with DAB Kit (Golden Bridge International, Inc., Bothell, WA, USA).

### Quantification of tumor blood vessels in brain metastases

Microvessel density (MVD) in the metastatic lesions in brain was assessed by IHC with rat anti-mouse CD31 mAb (BD Biosciences, San Jose, CA, USA). MVD (%) was calculated from the ratio of the CD31-positive staining area to the total observed brain metastasis area in the viable lesion. Positive staining areas were calculated using the imaging analysis software (Definiens Tissue Studio®; Definiens AG, Munich, Germany).

### Statistical analysis

Differences between two groups were assessed using Student’s *t*-test, or Wilcoxon’s rank sum test. All reported *p* values were two-sided, and those < 0.05 were considered to be statistically significant. Data are represented as mean and SD. All statistical analyses were conducted using the JMP® Version 11 software (SAS Institute, Inc., Cary, NC, USA).

## **Results**

### **Correlation of Nluc activity and TV**

**Fig. 1:**
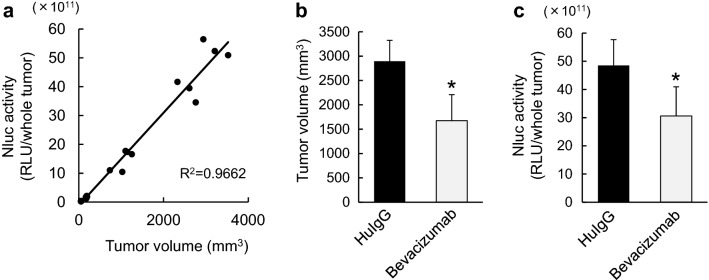
The correlation of tumor volume with NanoLuc® activity in Nluc-H1915 tumors grown subcutaneously in xenografted mice.** a** SCID mice were subcutaneously implanted with Nluc-H1915 cells, and on days 8, 14, 32 and 35, mice in groups of 5, 5, 5, and 6 (total of 21 mice) were respectively selected and subjected to measurement for tumor volume and Nluc activity (RLU/whole tumor) in the supernatant of tumor homogenate. The graph displays the relationship between tumor volume and Nluc activity. Each dot in **a** represents data from a single tumor grown in a single mouse. R^2^ is the coefficient of determination for linear regression analysis of data. **b** and** c** Mice bearing Nluc-H1915 xenografts received 5 mg/kg of bevacizumab or HuIgG as control intraperitoneally on days 1, 8, and 15 (n = 6/each group). The antitumor activity was evaluated on day 22 by measuring TV (**b**) and Nluc activity (RLU/whole tumor) in the supernatant of homogenized tumor (**c**). Bars represent mean + SD. *p < 0.05 versus HuIgG-treated group on day 22 (assessed by the Wilcoxon test)

To develop a useful method of determining the total amount of brain metastases and the antitumor activity of a drug on established brain metastases, we first assessed the correlation between TV and Nluc activity in a subcutaneous xenograft mouse model using Nluc-H1915 cells. When Nluc activity in Nluc-H1915 tumors was measured on days 8, 14, 32 and 35 in SCID mice subcutaneously inoculated with Nluc-H1915 cells, the Nluc activity significantly correlated with TV (Fig. 1a), with a high coefficient of determination (R^2^) value. Next, we evaluated the inhibitory effect of bevacizumab on the growth of Nluc-H1915 cells, and a similar association was observed between TV and Nluc activity in the tumor; that is to say, the mean TV in SCID mice treated with bevacizumab for 22 days was significantly lower than in control mice that received HuIgG (Fig. 1b) and similarly, mean Nluc activity in bevacizumab-treated tumors was also significantly lower than in control mice (Fig. 1c). These results indicate that the tumor-growth inhibitory activity of bevacizumab can be evaluated by measuring Nluc activity in this model.

### Profile of the hematogenous brain metastasis model

**Fig. 2:**
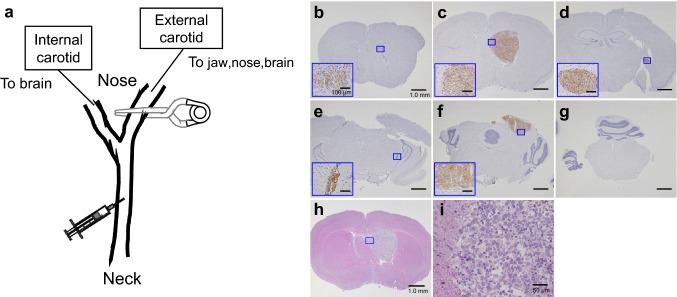
Metastasis of Nluc-H1915 established in brain. **a** Nluc-H1915 cell transplantation into the internal carotid artery of SCID mice, with the external carotid artery microclamped. **b–g** Micrographs of brain slices from the same mouse that include metastatic Nluc-H1915 tumor nodules. They were stained by IHC using an anti-human EGFR antibody with hematoxylin for counter staining. Brain samples were collected on day 32 or 37 after Nluc-H1915 inoculation and sliced into six sections, each 2 mm thick. Scale bars are 1.0 mm (or 100 µm at higher magnification). **h** and **i** Representative micrographs at low and high magnification, respectively, of the same brain slide adjacent to that shown in **c**, which contains a metastatic nodule in the parenchyma stained by HE. Scale bars are 1.0 mm and 50 µm in **h** and **i**, respectively

The schema in Fig. 2a depicts the position of the microclamp in the hematogenous model. In the preliminary experiment, brain metastasis was confirmed by microscope in the slices taken from inoculated mice. As shown in Fig. 2b–g, Nluc-H1915 cells expressing human EGFR protein can be distinguished from mouse brain parenchymal cells by IHC. Figure 2h, i show the micrographs of the same slice with HE staining at low and high magnification, respectively. In the center of Fig. 2h, a nodule with clearly different features from brain parenchyma can be seen, and Fig. 2i shows that the nodule consists of random-oriented and irregular-shaped cells. Micrographs of the section adjacent to that shown in Fig. 2h also depict the nodule, in which most of the cells showed positive for human EGFR (Fig. 2c). These results confirm the viability of the hematogenous brain metastasis model using Nluc-H1915 cells.

Human VEGF, which is believed to be derived from Nluc-H1915 cells, was detected in the brain homogenate at a mean concentration of 2.91 ± 0.67 ng/whole brain (mean ± SD, N = 9) by a specific enzyme immunoassay for human VEGF.

### The effects of bevacizumab on the brain metastasis model

**Fig. 3:**
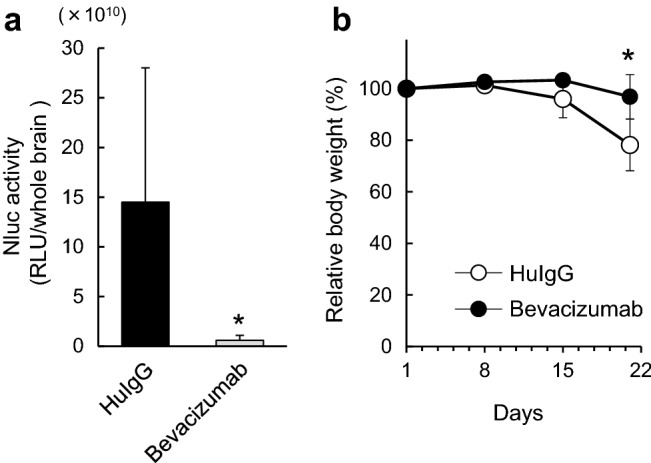
Antitumor effects of bevacizumab on established brain metastasis. Bevacizumab or HuIgG (5 mg/kg) was administered intraperitoneally into brain metastasis model mice or normal mice on days 1, 8 and 15 (n = 7/each group). **a** Antitumor activity of bevacizumab was evaluated by measuring the Nluc activity (RLU/whole brain) in the supernatant of brain homogenites on day 21. Bars represent mean + SD. *p < 0.05 versus HuIgG (assessed by the Wilcoxon test). **b** Relative body weight of mice given bevacizumab or HuIgG. Bars represent mean ± SD. *p < 0.05 versus HuIgG (assessed by the Student’s *t*-test)

The inhibitory effect of bevacizumab on brain metastasis was determined by comparing Nluc activity in the brains of bevacizumab- and HuIgG-treated mice. The Nluc activity in the bevacizumab-treated group was significantly reduced (Fig. 3a), which indicates the anti-proliferative effect of bevacizumab on the brain metastases in this model. Figure 3b depicts the time courses of relative body weight changes in the control and the bevacizumab-treated mouse groups. The change in the control mice reached approximately 20%, which was significantly greater than in bevacizumab-treated mice.

### Distribution of bevacizumab in the metastatic brain tumor

**Fig. 4:**
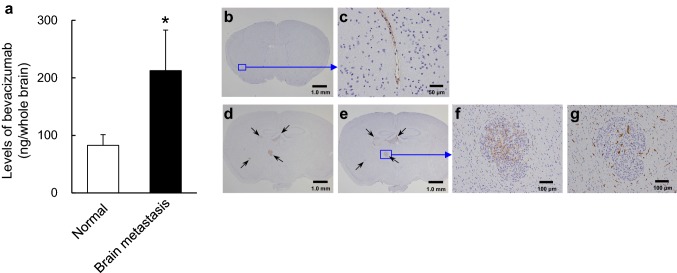
Distribution of bevacizumab in brain metastatic lesions. Bevacizumab (5 mg/kg) was administered intraperitoneally into mice with brain metastasis or normal mice. Brains were removed from the mice on days 20–22. **a** Levels of bevacizumab, determined by human IgG ELISA, in the brain of metastatic mice. Bars represent mean + SD. *p < 0.05 versus normal (assessed by the Wilcoxon test, n = 7/group). **b–g** Brain slices were stained by IHC using anti-human IgG antibody (for bevacizumab) and anti-human EGFR antibody (for tumor cells), anti-CD31 antibody with hematoxylin for counterstaining. Representative IHC images are shown in **b–g**. **b** and **c S**ame slice of brain from a normal mouse treated with bevacizumab. Scale bars are 1 mm and 50 µm in **b** and **c**, respectively. **d**–**g** Adjacent slices of brain from a mouse with brain metastasis treated with bevacizumab. **d** Brain section stained with an anti-human EGFR antibody. Scale bar is 1 mm. **e** (Lower magnification) and **f** (higher magnification) show a brain section stained with anti-human IgG to detect bevacizumab. Scale bars are 1 mm and 100 µm in **e** and **f**, respectively. **g** (Higher magnification) shows a brain section stained with CD31. Scale bar represents 100 µm

To evaluate the distribution of bevacizumab in metastatic brain tumors, we compared the levels of bevacizumab in the brain homogenates of normal mice and metastasis mice treated with bevacizumab. The results of the ELISA assay (which used an anti-HuIgG antibody to detect bevacizumab) showed that bevacizumab levels in the metastasis mice were significantly higher than in the normal mice (Fig. 4a), suggesting that bevacizumab can penetrate into the metastatic tumor site in the brain.

To clarify the distribution of bevacizumab in metastatic brain lesions, normal SCID mice and brain metastasis model mice were treated with bevacizumab and then their brain sections were stained by IHC using HuIgG to detect bevacizumab levels. As shown in Fig. 4b, c, bevacizumab (stained as HuIgG) was detected only in the microvessels of brain tissue in the normal mice. On the other hand, bevacizumab stained as HuIgG specifically penetrated into metastatic brain lesions consisting of Nluc-H1915 cells stained with EGFR (Fig. 4e, f), but it did not merge with CD31 stained in the brain metastasis model (Fig. 4g).

### MVD in the metastatic brain lesions of mice treated with bevacizumab

**Fig. 5:**
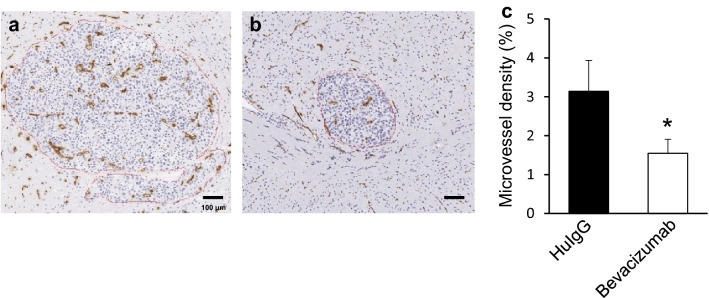
Effect of bevacizumab on MVD in the tumor area of parenchyma. 5 mg/kg of HuIgG (n = 9/group) or bevacizumab (n = 5/group) were administered intraperitoneally in the brain-metastasis model mice. Brains were removed from the mice on days 20 to 22 and sliced. The slices were stained by CD31 IHC to determine MVD in the tumor area of parenchyma. **a** and **b** Tumor vessels stained by IHC with anti-CD31 antibody in the brain metastases of HuIgG- and bevacizumab-treated mice, respectively. Scale bar represents 100 µm. **c** Blocked and open bars are levels of MVD in the tumor area of HuIgG- and bevacizumab-treated mice, respectively. Bars represent the mean + SD. *p < 0.05 versus HuIgG-treated group (assessed by the Wilcoxon test)

Representative IHC images of brain sections prepared from HuIgG- and bevacizumab-treated mice are shown in Fig. 5a and b, respectively. Bevacizumab administration significantly decreased the MVD in brain metastases (Fig. 5c).

## **Discussion**

In this study, we demonstrated the anti-proliferative effect of bevacizumab on established brain metastases in a hematogenous brain metastasis xenograft model using the human NSCLC cell line, Nluc-H1915. We used NCI-H1915 cells to develop this xenograft model because they were originally isolated from a brain metastasis in a patient with NSCLC, and tumor spheres of these cells have been reported to form multifocal brain tumors when intracranially injected into NOD-SCID mice [5, 22]. Of note, we chose to inject the tumor cells through the internal carotid artery in these mice, because the intracarotid artery injection of tumor cells recreates the latter part of hematogenous brain metastasis, e.g. adhesion to the brain vasculature, extravasation, and outgrowth in a brain-specific environment that supplies oxygen and nutrients [9], while clamping the external carotid artery is expected to further reduce metastases to other organs. Because these characteristics reflect the clinical setting for NSCLC patients with brain metastases, this model is suitable for practically evaluating the antitumor effect of bevacizumab on established brain metastases. In addition, this method can also be used to investigate earlier steps—when metastatic nodules form in the vascular niche—and for evaluating the drug’s effect on adhesion to the brain vasculature and extravasation.

It is difficult to quantify brain metastases, especially when they have a multifocal profile. The Nluc-H1915 cell line established in this study stably expresses the Nluc reporter gene and can also be transplanted into SCID mice. Nluc activity in the lysates of tumors grown in these mice correlated significantly with TV at a high R^2^ value, and the antitumor activity of bevacizumab in mice subcutaneously transfected with Nluc-H1915 could be assessed. Thus, Nluc-H1915 cells allowed us to estimate brain metastasis tumors semi-quantitatively, using a lysate of the whole brain parenchyma including metastases.

Regimens that contain bevacizumab are recommended as standard first-line therapy for metastatic NSCLC. Bevacizumab has recently been reported to have encouraging efficacy in patients with NSCLC who have asymptomatic untreated brain metastases, as shown by the phase 2 BRAIN study, with activity shown in both extracranial and intracranial lesions [1, 10, 11, 23]; however, its penetration into brain lesions and the mode of action have not yet been fully investigated. Specific features of the BBB have been reported to affect systemic treatment of metastatic tumors in the brain [24, 25], since hydrophilicity and the large molecular size of an antibody are considered disadvantageous for penetration. Tumor cells injected through the internal carotid artery in our mouse model reproducibly formed metastatic nodules in the brain, which theoretically suggests that the character of the BBB in this model is similar to those of patients with brain metastasis. The mean concentration of bevacizumab in the brain parenchyma was higher in the brain metastasis model mice than in normal mice (Fig. 4a), and when assessed by IHC, bevacizumab localized inside blood vessels in the normal brain parenchyma of metastatic mice as well as in normal mice. However, it did specifically penetrate into the brain metastatic lesions (EGFR+) caused by hematogeneous metastases, which is absolutely required for this antibody to inhibit the extravascular VEGF produced by tumor cells. As a result of this intratumoral penetration, bevacizumab worked well in reducing MVD. This suggests that bevacizumab could penetrate into tumor tissues because the BBB in the metastatic brain lesions is altered, whereas in normal mice it is intact. Indeed, a study by Oosting et al. of PET scans of patients with metastatic renal cell carcinoma given ^89^Zr-bevacizumab suggested that bevacizumab accumulates in metastatic brain lesions but not in normal brain parenchyma [26], though it was uncertain whether there was enough accumulated bevacizumab to provide efficacy. Taking our result and this report together, it is highly likely that the clinical efficacy of bevacizumab in patients with NSCLC with brain metastases reported in the BRAIN and the EOLE studies [10, 11] was achieved because bevacizumab was able to penetrate the altered BBB and enter metastatic lesions.

Recent studies have reported the efficacy of other therapeutic antibodies that inhibit immune checkpoints in patients with asymptomatic brain metastases [27, 28], which provides further evidence for the penetration of IgG into metastatic brain tissue and supports our findings. The precise status of BBB in metastatic brain nodules and the mechanism of penetration and/or transportation of IgG across the BBB should be investigated further, both clinically and in preclinical studies.

Bevacizumab inhibits VEGF-induced neovascularization, which is required for tumor growth, and partially normalizes the abnormal hyper-permeability caused in tumor vessels by VEGF-A. This normalizing effect decreases interstitial fluid pressure in the tumor and improves drug delivery into tumor tissues [29]. In this study, Nluc-H1915-derived VEGF was detected in the brain, bevacizumab was found distributed in brain metastatic lesions (Fig. 4d–f), and MVD in the brain metastasis was significantly lower in mice treated with bevacizumab than in HuIgG-treated mice (Fig. 5c). The inhibition of neovascularization appears to be at least one of the major modes of action for the anti-proliferative effect of bevacizumab on brain metastases in this model.

In conclusion, we demonstrated the anti-proliferative effect of bevacizumab on established brain metastasis in the hematogenous brain metastasis xenograft model. The distribution of bevacizumab in metastatic lesions suggests that it penetrated the BBB, and the reduction in MVD suggests that it suppressed angiogenesis, which indicates that the anti-proliferative effect of bevacizumab on brain metastasis observed in this study is due to its anti-angiogenic activity. The mechanism by which HuIgG penetrates the BBB in the hematogenous metastatic model and/or in patients with brain metastasis needs to be elucidated in more detail. The outcomes of this study suggest that bevacizumab has efficacy against established brain metastases, and that a regimen containing bevacizumab could be a promising treatment option for patients with NSCLC with brain metastases.

## Abbreviations

BBB: Blood–brain barrier
CNS: Central nervous system
ELISA: Enzyme-linked immunosorbent assay
GBM: Glioblastoma multiforme
HE: Hematoxylin and eosin
HuIgG: Human immunoglobulin G
IHC: Immunohistochemistry
mAb: Monoclonal antibody
MVD: Microvessel density
NSCLC: Non-small-cell lung cancer
RLU: Relative light unit
TV: Tumor volume
VEGF: Vascular endothelial growth factor
